# The Reaction N(^2^D) + CH_3_CCH (Methylacetylene): A Combined Crossed Molecular Beams and Theoretical Investigation and Implications for the Atmosphere of Titan

**DOI:** 10.1021/acs.jpca.1c06537

**Published:** 2021-10-05

**Authors:** Luca Mancini, Gianmarco Vanuzzo, Demian Marchione, Giacomo Pannacci, Pengxiao Liang, Pedro Recio, Marzio Rosi, Dimitrios Skouteris, Piergiorgio Casavecchia, Nadia Balucani

**Affiliations:** † Dipartimento di Chimica, Biologia e Biotecnologie, Università degli Studi di Perugia, 06123 Perugia, Italy; ‡ Dipartimento di Ingegneria Civile e Ambientale, Università degli Studi di Perugia, 06125 Perugia, Italy; § Master-Tec srl, Via Sicilia 41, 06128 Perugia, Italy

## Abstract

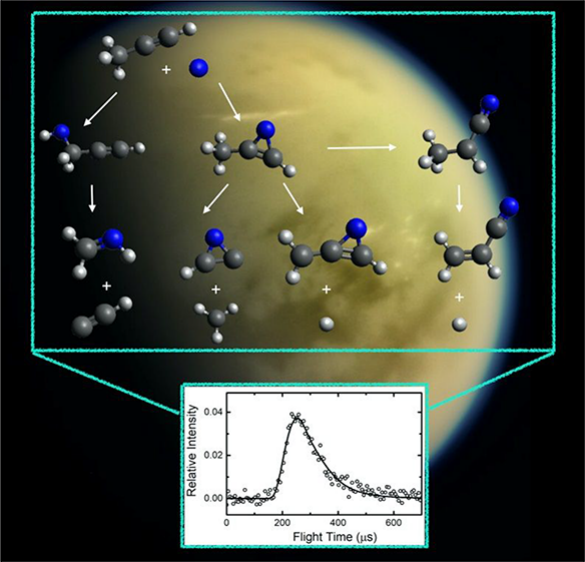

The reaction of excited nitrogen atoms N(^2^D) with CH_3_CCH (methylacetylene) was investigated under single-collision conditions by the crossed molecular beams (CMB) scattering method with mass spectrometric detection and time-of-flight analysis at the collision energy (*E*_c_) of 31.0 kJ/mol. Synergistic electronic structure calculations of the doublet potential energy surface (PES) were performed to assist the interpretation of the experimental results and characterize the overall reaction micromechanism. Theoretically, the reaction is found to proceed via a barrierless *addition* of N(^2^D) to the carbon–carbon triple bond of CH_3_CCH and an *insertion* of N(^2^D) into the CH bond of the methyl group, followed by the formation of cyclic and linear intermediates that can undergo H, CH_3_, and C_2_H elimination or isomerize to other intermediates before unimolecularly decaying to a variety of products. Kinetic calculations for addition and insertion mechanisms and statistical (Rice-Ramsperger-Kassel-Marcus) computations of product branching fractions (BFs) on the theoretical PES were performed at different values of total energy, including the one corresponding to the temperature (175 K) of Titan’s stratosphere and that of the CMB experiment. Up to 14 competing product channels were statistically predicted, with the main ones, at *E*_c_ = 31.0 kJ/mol, being the formation of CH_2_NH (methanimine) + C_2_H (ethylidyne) (BF = 0.41), *c*-C(N)CH + CH_3_ (BF = 0.32), CH_2_CHCN (acrylonitrile) + H (BF = 0.12), and *c*-CH_2_C(N)CH + H (BF = 0.04). Of the 14 possible channels, seven correspond to H displacement channels of different exothermicity, for a total H channel BF of ∼0.25 at *E*_c_ = 31.0 kJ/mol. Experimentally, dynamical information could only be obtained about the overall H channels. In particular, the experiment corroborates the formation of acrylonitrile + H, which is the most exothermic of all 14 reaction channels and is theoretically calculated to be the dominant H-forming channel (BF = 0.12). The products containing a novel C–N bond could be potential precursors to form other nitriles (C_2_N_2_, C_3_N) or more complex organic species containing N atoms in planetary atmospheres, such as those of Titan and Pluto. Overall, the results are expected to have a potentially significant impact on the understanding of the gas-phase chemistry of Titan’s atmosphere and the modeling of that atmosphere.

## 1 Introduction

Titan is a massive moon of Saturn. Mostly composed of rocks (60%) and water ice (40%), its atmosphere has triggered the attention of the scientific community starting with the detection of CH_4_ by Kuiper^1^ in 1944, as a somewhat rich organic chemistry based on the photochemistry of methane was speculated. After several exploration missions (Pioneer 11, the two Voyagers, and, much later, the amazing Cassini-Huygens mission) and ground-based observations (even by means of the Atacama Large Millimeter Array (ALMA) interferometer in recent years^2^) our knowledge of Titan and its unique atmosphere has reached an unprecedented level. Voyager missions revealed that molecular nitrogen is by far the main component,^3,4^ with methane accounting only for a few percent. At the same time, however, the detection of nitriles in trace amounts of a few parts per billion increased the interest for the atmospheric chemistry of this peculiar moon,^5^ because they are considered crucial precursors of biomolecules.^6−8^ The Cassini-Huygens mission confirmed the richness of Titan atmospheric chemistry by verifying all the previous observations and allowing the detection of more organic molecules and ions in the thermosphere of Titan.^9,10^ But the organic chemistry of Titan goes much further. Since the Voyager missions, we know that Titan is completely covered by a thick orange haze, the origin of which has challenged the scientific community. The data collected by the Huygens aerosol analyzer during its descent indicate that the aerosols are nitrogen-rich organic macromolecules.^11^ Finally, the icy surface of the moon is covered by solid deposits of organic compounds (e.g., benzene, cyanoacetylene, and acetylene), and lakes of liquid methane/ethane are present.^12,13^ Methylacetylene (propyne) has been among the first hydrocarbons to be detected by the Voyager mission.^5,14,15^ Together with its structural isomer allene (propadiene), it is predicted by all photochemical models to be formed in the upper atmosphere of Titan by the chemistry initiated by methane photodissociation. However, the detection of allene has become possible only recently with the first unambiguous detection by Lombardo et al.^16^ in 2019 by means of the Texas Echelle Cross Echelle Spectrograph (TEXES) mounted on the National Aeronautics and Space Administration (NASA) Infrared Telescope Facility. The allene derived abundance at 175 km is (6.9 ± 0.8) × 10^–10^ mole fraction, with an abundance ratio of methylacetylene over allene of 8.2 ± 1.1 at 150 km.

As already mentioned, in addition to hydrocarbons, nitriles have also been detected, while the aerosol organic macromolecules contain a large fraction of nitrogen. Clearly, N-active species must be also formed in the upper atmosphere of Titan because there are no organic radicals that can react with ground-state N_2_ at typical temperatures of Titan’s atmosphere. The role of nitrogen atoms or ions (both N^+^ and N_2_^+^) as well as electronically excited states of N_2_ (as the metastable *A*^3^Σ^+^_u_) have been considered in all photochemical models. Atomic nitrogen can be formed by an extreme ultraviolet photolysis of N_2_ but also by N_2_ dissociative photoionization, dissociation via an electron impact, galactic cosmic ray absorption, and N_2_^+^ dissociative recombination.^17−19^ All these processes can lead to the production of atomic nitrogen in its ground ^4^S_3/2_ state but also in its first electronically excited ^2^D_3/2,5/2_ states. The ^2^D_3/2,5/2_ states are metastable with long radiative lifetimes (13.6 and 36.7 h for ^2^D_3/2_ and ^2^D_5/2_, respectively),^20^ and, once formed in the upper atmosphere of Titan above 800 km from the surface, the main destiny of N(^2^D) is to react with other constituents of Titan’s atmosphere, since its collisional deactivation by N_2_ is not efficient. Clearly, the reactions of N(^2^D) with the hydrocarbons identified in the atmosphere of Titan can provide an efficient route toward the formation of nitriles and other N-rich organic molecules. As a matter of fact, little was known about N(^2^D) reactions until recently. Reliable laboratory experiments became available only in the late 1990s, because of the experimental difficulties in generating excited nitrogen atoms. The rate coefficients for the reactions N(^2^D) + CH_4_ and N(^2^D) + C_2_H_2_ have been investigated in a temperature range between ca. 220 and 290 K (for a critical review of those data see ref (17)). The kinetics of a few other N(^2^D) reactions were also investigated at room temperature or slightly lower temperature (*T*), but these were outside the range of relevance for Titan. The situation has recently changed as new results on the low-*T* kinetics for the reactions with CH_4_, C_2_H_6_, C_3_H_8_, C_2_H_2_, and C_2_H_4_ have been finally obtained in a range of temperatures encompassing those of relevance for Titan by means of the reaction kinetics in uniform supersonic flow (CRESU) technique.^21−23^ In addition to that, a systematic investigation of N(^2^D) reactions with simple hydrocarbons was undertaken by means of the crossed molecular beam (CMB) technique with mass-spectrometric detection by some of the present authors. CMB results have always been complemented by electronic structure calculations of the relevant stationary points of the underlying potential energy surface (PES) and statistical (Rice-Ramsperger-Kassel-Marcus) calculations of the branching fractions. This combined theoretical and experimental approach was applied to the multichannel reactions N(^2^D) + CH_4_, C_2_H_2_, C_2_H_4_, and C_2_H_6_.^24−28^ Much more recently, within the framework of the Italian National Project of Astrobiology,^29^ we conducted a combined experimental and theoretical investigation of the reaction of N(^2^D) with HCCCN (cyanoacetylene),^30^ C_5_H_5_N (pyridine),^31^ allene (unpublished results), C_6_H_6_ (benzene),^32−34^ and C_6_H_5_CH_3_ (toluene).^35^ In all cases, new species holding a novel C–N bond were identified as major reaction products, thus implying that N(^2^D) reactions with hydrocarbons are major players in the initiation of nitrile chemistry.

In this manuscript, we report on a combined experimental and theoretical investigation of the reaction N(^2^D) + CH_3_CCH. Specifically, we employed the CMB technique with a time-of-flight (TOF) analysis to explore the reaction mechanism and performed dedicated electronic structure calculations of the underlying PES. In addition, kinetic/RRKM estimates of the product branching fractions are reported. The information so obtained is expected to be useful in improving photochemical models of Titan’s atmosphere.^36−42^ A comparison with the results for the allene reaction can be of interest in understanding the chemistry of structural isomers as recently done for the reactions O(^3^P) + CH_3_CCH/CH_2_CCH_2_.^43^ The reactions between N(^2^D) and both methylacetylene and allene have already been included in photochemical models with estimated rate coefficients and product branching fractions. In the case of the N(^2^D) + CH_3_CCH reaction, by analogy with similar reactions Loison et al.^41^ suggested that the main reaction channels are those leading to HCCN + CH_3_ and C_2_H_3_CN (acrylonitrile) + H with a comparable yield. As a matter of fact, according to the present B3LYP/CCSD(T) calculations, there are many additional open reactive channels correlating with the reactants
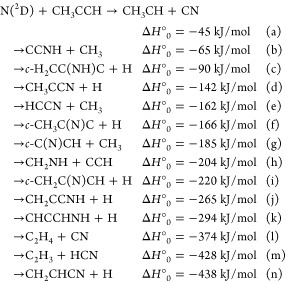
where the enthalpies of reaction reported are those calculated in the present work at the CCSD(T) level (see below).

The paper is organized as follows. In section 2 the experimental and theoretical methods are presented. The experimental results and the analysis of them are given in section 3, while the theoretical results are presented in section 4. A discussion follows in section 5, while section 6 provides the summary of the conclusions.

## 2 Experimental and Theoretical Methods

### 2.1 Experimental Method

The CMB apparatus has been described in some detail in our previous works, including a recent improvement related to the ion detection system.^44−47^ Briefly, two continuous supersonic beams of the reactants are crossed at a fixed angle of 90° in a large scattering chamber kept at 2 × 10^–6^ mbar during operation, which ensures single-collision conditions. After the collision, the reaction products are detected as a function of the laboratory (LAB) scattering angle or as a function of their velocity at selected LAB scattering angles, using a rotable electron impact quadrupole mass spectrometer detector and TOF analysis. The LAB angle Θ = 0° corresponds to the atomic nitrogen beam direction.

The atomic nitrogen beam was produced in a supersonic radio frequency (RF) discharge beam source^48,49^ by discharging 250 W of RF power on a 2.5% N_2_/He gas mixture at 125 mbar, through a 0.48 mm diameter water-cooled quartz nozzle, followed by a 0.8 mm diameter boron nitride skimmer, an ion deflecting field, and a further defining aperture. The fraction of the molecular dissociation and the electronic state (^4^S, ^2^D, ^2^P) distribution of the N atoms in the beam were as in our recent study on N(^2^D) + pyridine;^31^ the resulting beam velocity and speed ratio were also similar (2253 m/s and 4.6, respectively). In previous experiments on N atoms with small hydrocarbons,^24−28^ we observed that the presence of N(^4^S) (72%) and N(^2^P) (7%) in the N beam did not affect our experiment, because rate constants related to the N(^4^S) and N(^2^P) reactions with saturated/unsaturated hydrocarbons are much smaller than those of N(^2^D), as in the case of N(^2^D) + methane,^24^ N(^2^D) + ethane,^27^ N(^2^D) + acetylene,^25,50^ and N(^2^D) + ethylene.^26,28^

The supersonic beam of methylacetylene was generated by expanding 400 mbar of neat gas (98% purity) through a 0.1 mm diameter stainless steel nozzle, followed by a 0.8 mm skimmer and a further collimating aperture. The peak velocity was measured to be 654 m/s with a speed ratio of 4.0. The resulting collision energy, *E*_c_, is 31.0 kJ/mol.

Product angular distributions were measured in the LAB system by modulating at 160 Hz the hydrocarbon beam for a background subtraction. Product TOF distributions were measured at selected LAB angles by the pseudorandom chopping method at 6 μs/channel. Quantitative information is obtained by moving from the LAB coordinate system to the center of mass (CM) one and extracting the differential cross section, *I*_CM_(θ,*E*′_T_), which is commonly factorized into the product of the translational energy distribution, *P*(*E*′_T_), and the angular distribution, *T*(θ), that is *I*_CM_(θ,*E*′_T_) = *T*(θ) × *P*(*E*′_T_).^44−46^ Specifically, the *T*(θ) and *P*(*E*′_T_) distributions are assumed, averaged, and then transformed to the LAB frame for a comparison with the experimental distributions, and the procedure is repeated until a satisfactory fit of the latter is obtained. The *T*(θ) and *P*(*E*′_T_) functions contain all the information about the reaction dynamics.

### 2.2 Theoretical Methods

**Figure 1:**
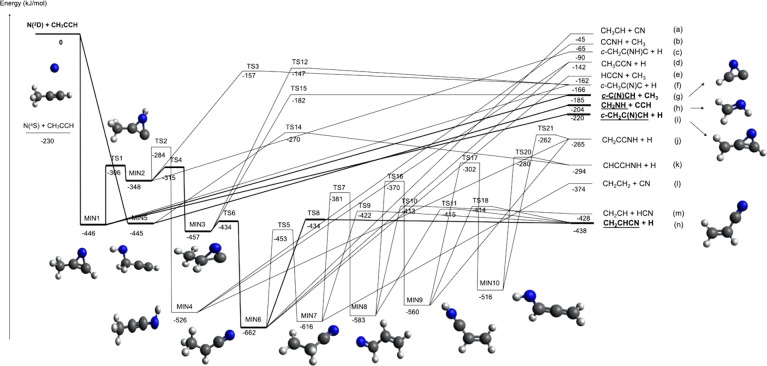
Schematic representation of the PES for the reaction N(^2^D) + CH_3_CCH with energies evaluated at the CCSD(T)/aug-cc-pVTZ level of theory (see text). The structure of the heavier coproduct from the four main product channels is shown as well as the structure of all intermediates. Heavier solid lines indicate the main pathways leading to the underlined four (statistically predicted) main products.

The potential energy surface for the reaction N(^2^D) + CH_3_CCH was characterized by adopting a computational procedure previously used for the analysis of several other reactions.^24−28,33,51−55^ In particular, the lowest stationary points were located at the B3LYP^56,57^ level of theory, in conjunction with the correlation consistent valence polarized set aug-cc-pVTZ.^58−60^ The same level of theory was utilized to compute the harmonic vibrational frequencies in order to check the nature of the stationary points, that is, minimum if all the frequencies are real, saddle point if there is one, and only one, imaginary frequency. The identified saddle points were assigned by performing Intrinsic Reaction Coordinates (IRC) calculations.^61,62^ The energy of all the stationary points were calculated at the higher level of calculation CCSD(T),^63−65^ with the same basis set aug-cc-pVT. Both the B3LYP and the CCSD(T) energies were corrected to 0 K by adding the zero-point energy correction computed using the scaled harmonic vibrational frequencies evaluated at the B3LYP/aug-cc-pVTZ level. The energy of N(^2^D) was estimated by adding the experimental^66^ separation N(^4^S)-N(^2^D) of 230.0 kJ/mol to the energy of N(^4^S) at all levels of calculation. All calculations were performed using Gaussian09,^67^ while analyses of the vibrational frequencies were performed using AVOGADRO.^68,69^ A schematic representation of the PES is shown in Figure 1, while reaction enthalpies and barrier heights for each reaction step evaluated at the CCSD(T)/aug-cc-pVTZ level of theory are given in Table S1 (Supporting Information).

### 2.3 Kinetic Calculations

The reaction N(^2^D) + CH_3_CCH was analyzed using RRKM theory,^70^ through a code implemented in our group for this purpose.^24,28^ The microcanonical rate constant *k*(*E*) for a specific reaction at a specific total energy is given by
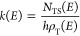
where *N*_TS_(*E*) is the sum of states at the transition state at energy *E*, *ρ*_T_(*E*) is the reactant density of states at energy *E*, and *h* is Planck’s constant.

As already illustrated in the previous section (see Figure 1), the reaction features two different initial approaches (insertion into one of the C–H bonds or addition to the triple bond) that are both barrierless. In cases like this, it is extremely difficult to predict the role of the two approaches, and only a full dynamical treatment can address this issue in a rigorous way.

Unfortunately, this kind of calculation is not feasible for systems of this complexity. Therefore, we decided to employ an approximate method. The initial bimolecular process has been described considering a potential *V* as a function of the distance *R* between the two particles: *V*(*R*) = −*C*_6_/*R*^6^. On the basis of this potential, capture (Langevin) theory^71^ was used to calculate the capture rate constant for the reactants as a function of energy. The assumption here is that the capture event precedes the selection between the addition or insertion intermediate. Thus, the rate coefficient calculated here represents the rate for the formation of a “captured” species whose fate (addition or insertion) has not been decided yet. The rate of formation of each of the two intermediates is calculated purely on the basis of statistics. In other words, the initial capture rate constant *k*_c_(*E*) is partitioned between the two intermediates based on their densities of states, *ρ*_add_(*E*) and *ρ*_ins_(*E*) according to the formula
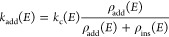
for the addition channel (and similar for the insertion channel). The assumption here is that the captured species can switch back and forth between the two intermediates before committing itself to one of them, and thus it is statistics that determines the outcome.

The rotational densities of states, for both the reactants and transition states, were calculated using an inverse Laplace transform of the corresponding partition functions. Subsequently, a convolution between the rotational densities of states and the corresponding vibrational ones was performed using a direct count algorithm. Finally, the sum of states was obtained by integrating the density of states with respect to energy. The effect of tunneling was taken into account by using the corresponding imaginary frequency of the transition state and calculating the tunneling probability for the corresponding Eckart barrier. In the dissociation channels without a clearly defined transition state, the variational RRKM approach was used. In particular, the results coming from several ab initio calculations, performed at various points along the reaction coordinate (with a step of 0.5–1.0 Å), were used for the RRKM analysis. The transition state for the channel is the point yielding the minimum value of the rate constant, in accordance with the variational approach (VTST).^72^ In the cases in which no intermediate points are available, due to difficulties in the electronic structure calculations, and the reaction channel has a dynamical exit barrier, as in our case, the transition state was assumed as the products at infinite separation.

## 3 Experimental Results

**Figure 2:**
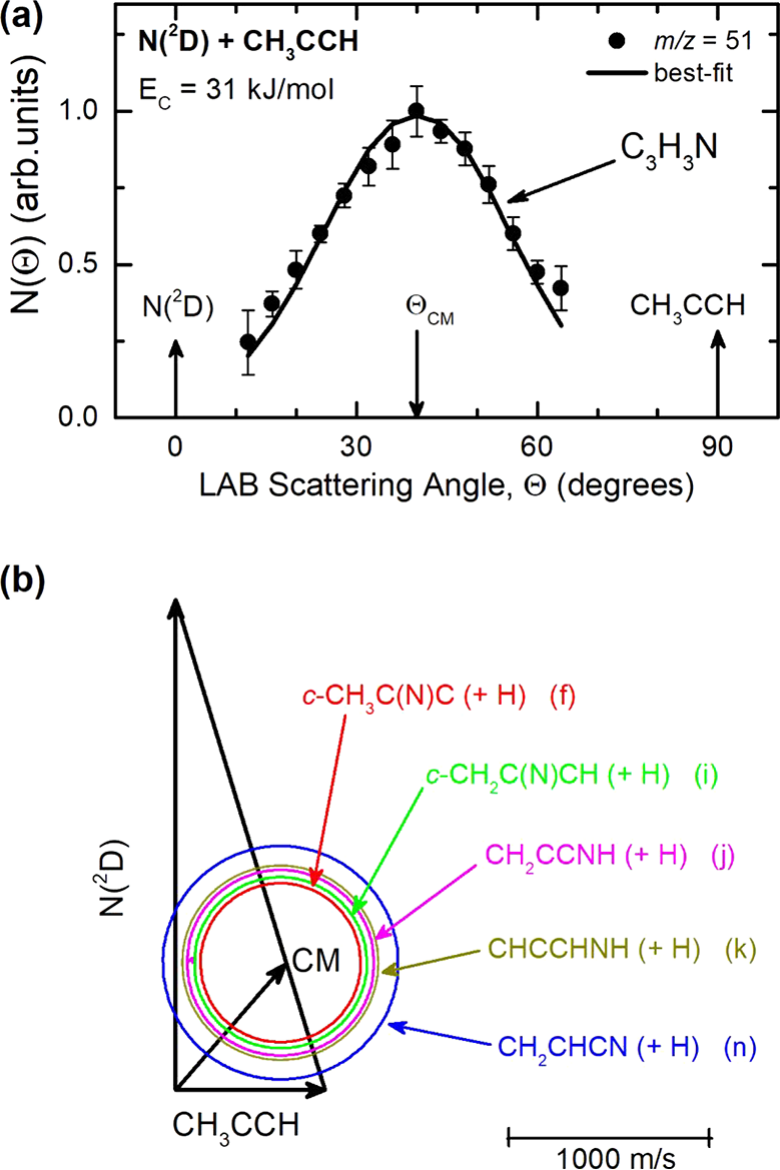
(a) LAB angular distribution at *m*/*z* = 51 (C_3_HN^+^) for the N(^2^D) + methylacetylene reaction at *E*_c_ = 31 kJ/mol. The solid blue curve represents the calculated distribution when using the best-fit CM functions shown in Figure 4. (b) Velocity vector (Newton) diagram of the experiment. The radius of each circle represents the maximum velocity that the indicated product can attain in the CM system if all available energy is channeled into product recoil energy. The labeling (f, i, j, k, and n) of the five indicated H forming channels is as in the text.

**Figure 3:**
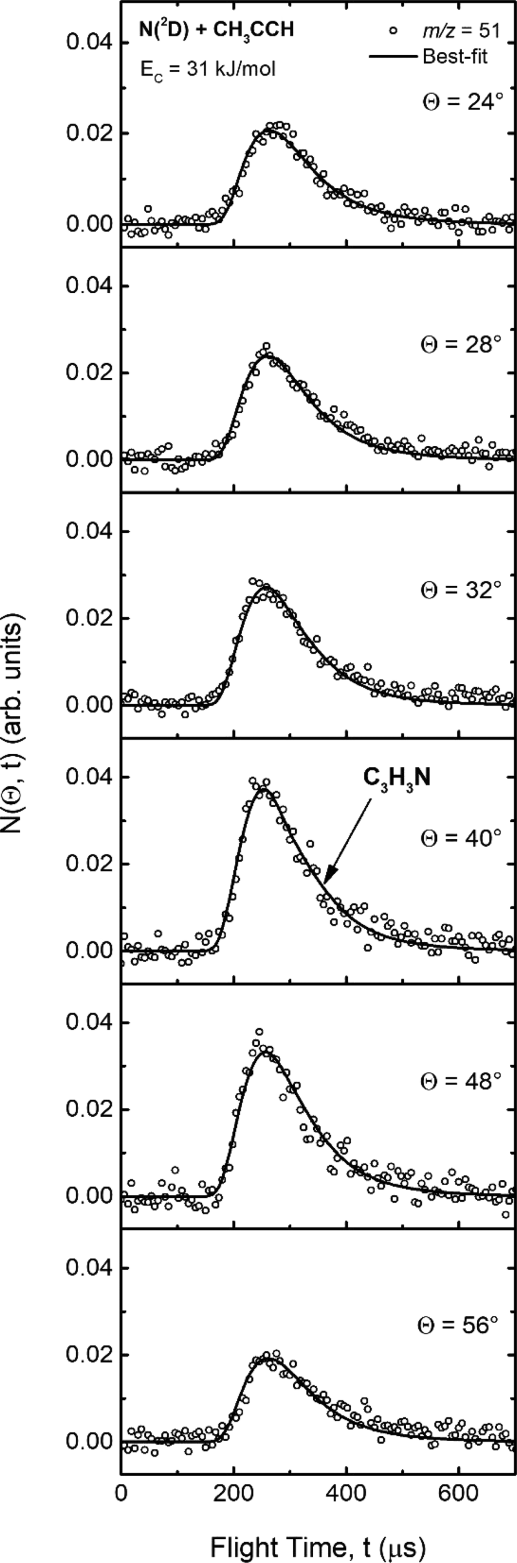
TOF distributions for *m*/*z* = 51 at angles Θ = 24°, 28°, 32°, 40°, 48°, and 56° for the N(^2^D) + methylacetylene reaction; the empty circles represent the experimental data, and the line shows the best-fit curves obtained with the CM functions shown in Figure 4.

A reactive scattering signal was observed at the following mass-to-charge ratios: *m*/*z* = 53, 52, and 51, with relative intensities of ∼0.2, 0.7, and 1.0, potentially corresponding to the H and H_2_ elimination channels. The angular distribution was registered using the hard electron ionization (70 eV) and accumulating the reactive signal for 50 s at each angle. The angular distributions at *m*/*z* = 53, 52, and 51 were identical, within the error bars (±1σ), indicating that all of them correspond to H-displacement channels. Because of the better signal-to-noise ratio (S/N), final measurements were performed at *m*/*z* = 51. Six scans at *m*/*z* = 51 were collected and averaged, corresponding to the ion with gross formula of C_3_HN^+^. The resulting product angular distribution is shown in Figure 2a, together with the velocity vector (Newton) diagram of the experiment (Figure 2b). The circles superimposed on the Newton diagram delimit the maximum velocity in the CM that the indicated products of the five most important, different H displacement channels n, j, f, k, and i (see below) can attain, assuming that all the available energy (given by *E*_c_ – Δ*H*^0^_o_) is channeled into the product translational energy. The circles also delimit the reactive scattering angular range in the LAB system for each product. The TOF distribution for *m*/*z* = 51 was measured at six different LAB angles and are shown in Figure 3. TOF distributions at the CM angle of 40° and at Θ = 24° were also measured for *m*/*z* = 53, which is the parent ion of the H displacement channel(s), and *m*/*z* = 52, which is the parent ion of a possible H_2_ elimination channel. Because also the TOF distributions were identical at *m*/*z* = 53, 52, and 51, it was concluded that a potential H_2_ elimination channel is negligible under our experimental conditions.

Notably, no signal was detected at *m*/*z* = 27 (within our sensitivity, i.e., branching fraction (BF) ≤ 0.02), ruling out the exoergic channel (1m) of the HCN formation. We also attempted to measure LAB distributions at *m*/*z* = 26 and 28 to characterize the channel (1l) leading to the formation of CN + C_2_H_4_. This attempt failed because of the strong interfering signals caused by undissociated molecular nitrogen from the atomic beam and the high inherent background due to CO in any ultrahigh vacuum (UHV) system. At *m*/*z* = 26 a small signal of the CN radical in a TOF distribution was obtained. However, this signal can be also originated by traces of CO_2_ contamination in the supersonic beam of N atoms, which reacts upon a radiofrequency discharge in the beam (CO_2_ + N(^2^D) → CN + O_2_). In fact, this *m*/*z* = 26 peak had a similar velocity to the elastically/inelastically scattered N atom beam at that LAB angle. For this reason, it was impossible to study this *m*/*z* channel.

Finally, according to the reaction scheme, the last possible products of N(^2^D) + CH_3_CCH would be the formation of CH_3_ (methyl radical) and, as coproducts, the three isomeric species with brute formula of C_2_NH (channels (b), (e), and (g)). Unfortunately, also in this case our experimental conditions did not allow us to demonstrate the presence of these species: indeed, at *m*/*z* = 39 (C_2_NH^+^) there was a very intense elastic signal due to the dissociative ionization of the CH_3_CCH reactant (parent ion *m*/*z* = 40), while at *m*/*z* = 15 (CH_3_^+^) no signal was observed in a TOF spectrum recorded at Θ_CM_ = 40°, with an ionization energy of 22 eV to prevent the contribution of ^15^N (in natural isotopic abundance) from the N beam, after 1 h of accumulation time. Note that the reactive signal, when using 22 eV of electron energy, reduces typically by a factor of 5–8 with respect to 70 eV, and therefore we could not put a lower limit for the fraction of the methyl-forming channels, which are kinematically strongly unfavored (by ∼2 orders of magnitude when detecting the light CH_3_ product, with respect to when detecting the heavy coproduct of an H-displacement channel). Therefore, we must rely on the RRKM statistical calculations for deriving an estimate of the BF of the possible CH_3_ forming channels (b), (e), and (g) (see section 4.2). A probe of channel (h) was not possible because of the very high detector background at *m*/*z* = 28 (see above), affecting also *m*/*z* = 29.

The angular distribution at *m*/*z* = 51 (Figure 2a) shows that the reactive signal extends beyond the explored 12°–64° LAB angular range. Although the angular distribution is centered around the CM angle of 40°, its width is rather large for an H displacement product channel. Indeed, the Newton circles around the CM angle in the velocity vector diagram (see Figure 2b) are rather large, and this is because several of the possible H-forming channels are very exothermic. Therefore, a wide angular distribution indicates that a large fraction of the total available energy is channeled into the product translation (see below). In Figure 3 the TOF distributions registered for *m*/*z* = 51 at six different angles (Θ = 24°, 28°, 32°, 40°, 48°, and 56°) are characterized by a single, fast, and rather broad peak centered at ∼250 μs.

**Figure 4:**
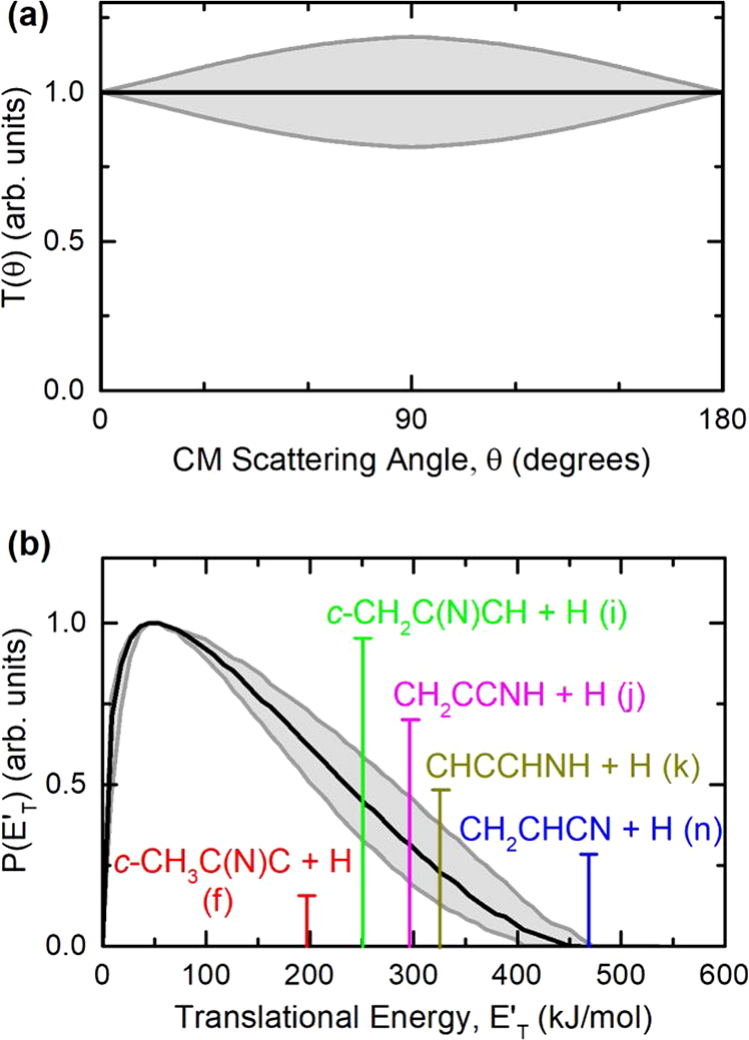
Best-fit product angular (a) and translational energy (b) distributions, *T*(θ) and *P*(*E*′_T_), in the CM system for the N(^2^D) + methylacetylene system. The shaded areas represent the error bars determined for the CM functions. The vertical lines in the graph of *P*(*E*′_T_) indicate the total energy (*E*_tot_ = *E*_c_ – Δ*H*^0^_0_) of five different isomers with formula of C_3_H_3_N, corresponding to the five most exothermic H forming channels (f), (i), (j), (k), and (n).

The continuous curves in the Figures 2a and 3 represent the best-fit using the CM functions portrayed in the Figures 4a,b. The data at *m*/*z* = 51 were fit using a single couple of *T*(θ) and *P*(*E*′_T_) functions. As Figure 4a shows, the best-fit angular distribution in the CM, *T*(θ), is isotropic throughout the angular range (within the error bars), and this is consistent with the formation of a long-lived complex (i.e., a complex living more than five to six rotational periods, according to the osculating complex model for chemical reactions^73,74^). Incidentally, this observation sustains the adoption of the RRKM statistical method for the kinetics estimates.

The *P*(*E*_T_′) exhibits a peak at ∼46 kJ/mol and extends up to ∼455 kJ/mol. The average product translational energy, defined as ⟨*E*_T_′⟩ = ∑*P*(*E*′_T_)*E*′_T_/∑*P*(*E*′_T_), is ∼150 kJ/mol and corresponds to a fraction, ⟨*f*_T_⟩ (⟨*f*_T_⟩ = ⟨*E*_T_′⟩/*E*_tot_), of the total available energy of 0.32 released in product translation, when using for *E*_tot_ (*E*_tot_ = *E*_c_ – Δ*H*^0^_0_) the theoretical value of the most exothermic H-displacement channel (n), leading to acrylonitrile (cyanoethylene) + H (Δ*H*^0^_0_ = −438 kJ/mol). Because the *P*(*E*′_T_) extends up to the limit of the most exothermic H-forming channel (n), this indicates that this channel is certainly occurring to a sizable fraction. However, we cannot rule out the occurrence of also the other, less exothermic H-forming channels (c, d, f, i, j, and k). To discriminate which C_3_H_3_N isomers are formed and estimate their BFs, we combine the experimental observation with the ab initio and RRKM statistical calculations (see next section).

## 4 Theoretical Results

### 4.1 Electronic Structure Calculations

The PES (see Figure 1) obtained for the reaction N(^2^D) + CH_3_CCH shows 10 different minima (MIN1, MIN2, MIN3, MIN4, MIN5, MIN6, MIN7, MIN8, MIN9, MIN10) linked by eight possible transition states (TS1, connecting MIN1 and MIN2; TS2, connecting MIN2 and MIN4, TS4, connecting MIN2 and MIN3; TS6, connecting MIN3 and MIN6; TS5, connecting MIN6 and MIN7; TS7, connecting MIN7 and MIN8; TS16, connecting MIN8 and MIN9; TS17, connecting MIN9 and MIN10).

**Figure 5:**
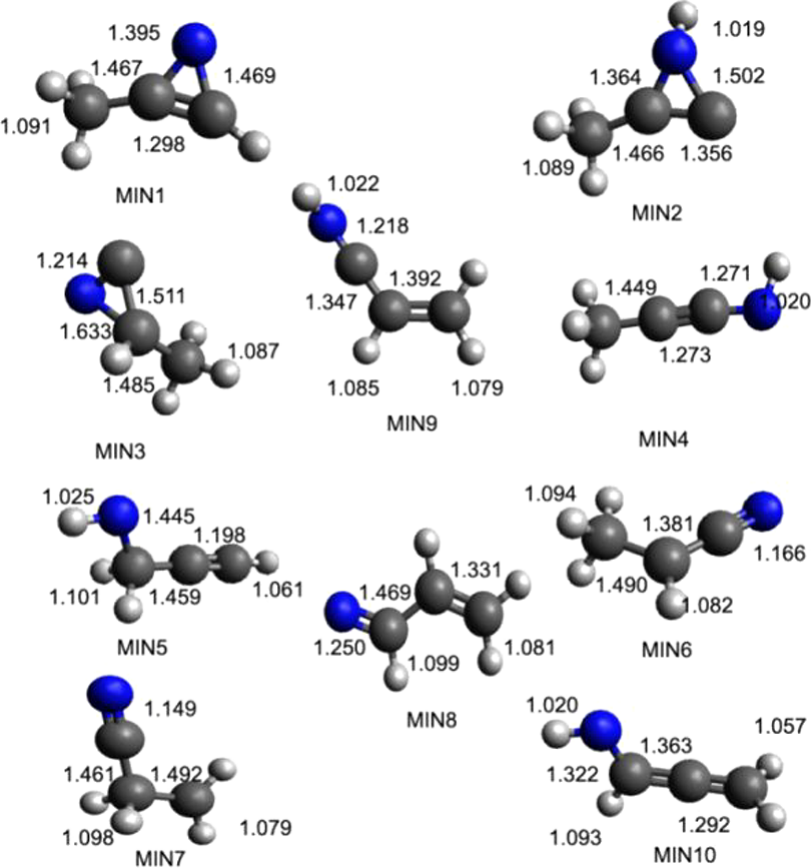
B3LYP optimized geometries (in Å) of the minima identified along the PES for the reaction N(^2^D) + CH_3_CCH.

**Figure 6:**
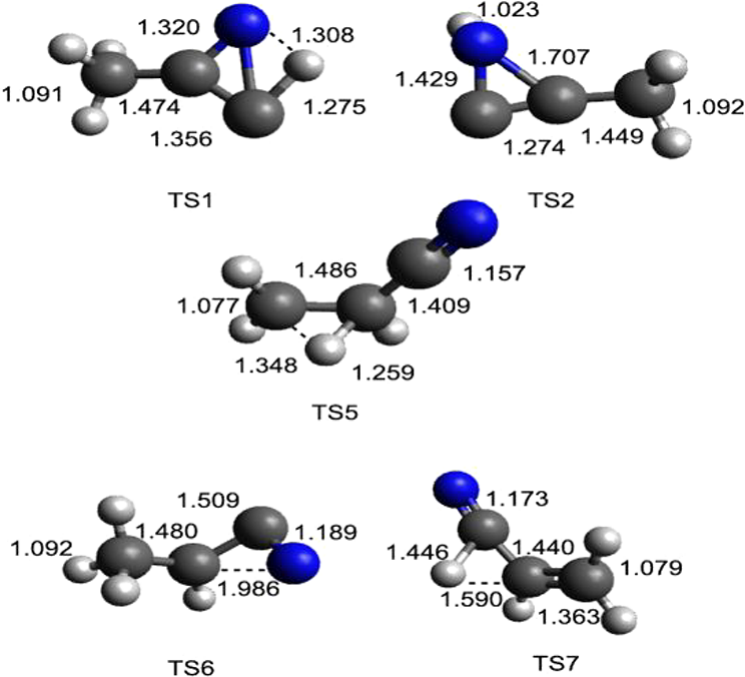
B3LYP optimized geometries (in Å) of the main transition states identified along the PES for the reaction N(^2^D) + CH_3_CCH.

**Figure 7:**
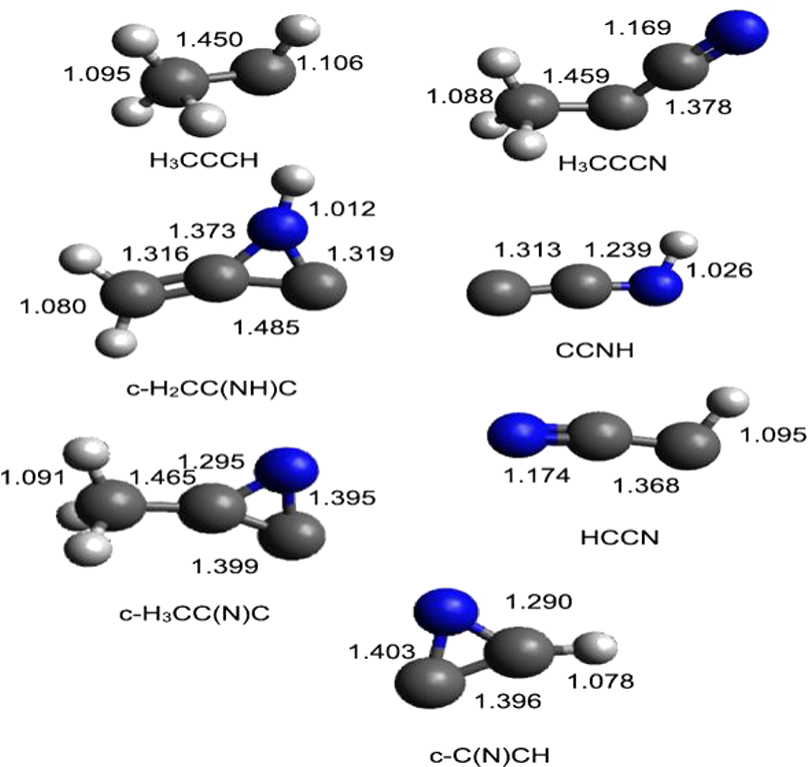
B3LYP optimized geometries (in Å) of the possible products identified along the PES for the reaction N(^2^D) + CH_3_CCH.

**Figure 8:**
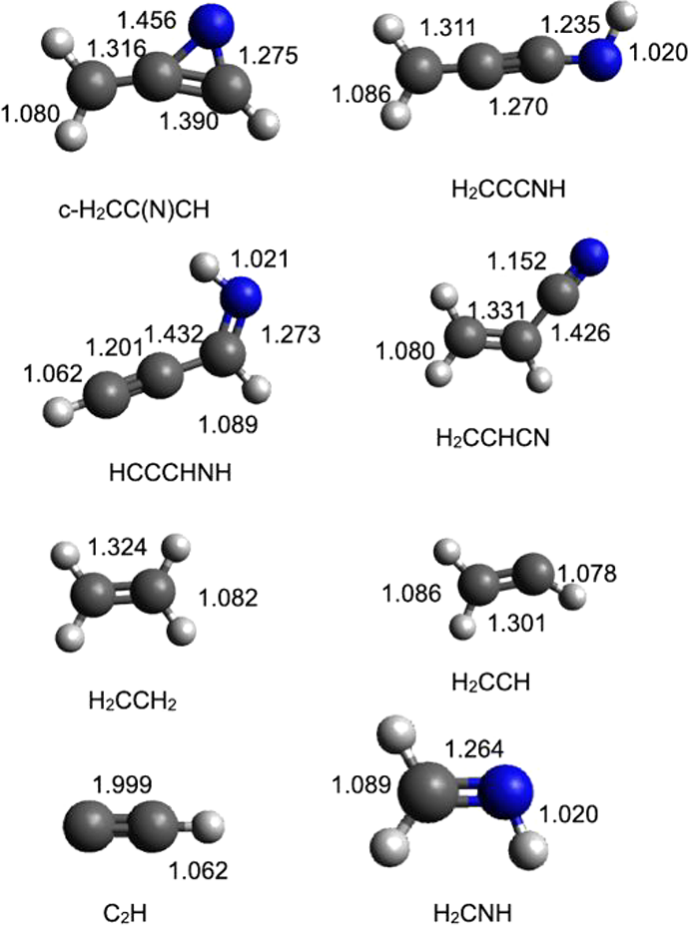
B3LYP optimized geometries (in Å) of the possible products identified along the PES for the reaction N(^2^D) + CH_3_CCH.

The addition of N(^2^D) to the triple bond of CH_3_CCH is barrierless and leads to the formation of the cyclic intermediate MIN1 (located 446 kJ/mol below the reactant energy asymptote). An alternative barrierless approach is represented by the insertion of N(^2^D) into one of the three C–H bonds of the CH_3_ group forming the intermediate MIN5. This last species can then undergo a dissociation via TS14 forming H and propargylimine (HCCCHNH) (channel k) with a global reaction enthalpy of −294 kJ/mol. The related transition state (TS14), which shows the breaking of a CH bond, is located 270 kJ/mol below the reactant energy asymptote. A different fate of MIN5 is represented by the fission of the C–C σ bond, which allows the formation of the products CCH (ethylidyne) and methanimine (H_2_CNH) (channel h) without an exit barrier (the reaction enthalpy for this channel is −204 kJ/mol). Several products can be formed starting from the aforementioned MIN1. The fission of a C–H bond of the CH_3_ group leads to the production of H and the cyclic cofragment *c*-H_2_CC(N)CH, with a reaction enthalpy of −220 kJ/mol (channel (i), while fission of the acetylenic C–H bond leads to formation of H + *c*-CH_3_C(N)C (with an exothermicity of 166 kJ/mol) (channel f). A third identified channel is related to the breaking of a C–C bond with the subsequent formation of CH_3_ + *c*-C(N)CH (channel g) with a reaction enthalpy of −185 kJ/mol. A barrier of 140 kJ/mol (TS1) must be overcome in order to form the isomer MIN2, in which a new N–H bond is formed. This last intermediate can dissociate, forming both H + *c*-CH_3_C(N)C (channel f) (also formed through the dissociation of MIN1, through a barrier of 191 kJ/mol) and H + *c*-CH_2_C(NH)C (channel c) (the resulting exothermicity is 90 kJ/mol) in a barrierless process. MIN3 can be formed via an isomerization of MIN2, overcoming a barrier (TS4) of 33 kJ/mol. The related transition state shows the breaking of the N–H bond and the subsequent formation of a new C–H bond. This last isomer can lead to two different barrierless product channels. The fission of a C–H bond leads to the formation of the previously described H + *c*-CH_3_C(N)C products (channel f), while the breaking of a C–C bond leads to a production of CH_3_, together with the aforementioned cofragment *c*-C(N)CH (channel g). Other isomerization processes can be invoked to explain the formation of linear intermediates. In particular, the ring opening of MIN2 can produce the MIN4 intermediate, which appears to be 178 kJ/mol more stable. The isomerization barrier (TS4) is 64 kJ/mol. A similar ring-opening mechanism, with a barrier of 23 kJ/mol, can form MIN6 (located at −662 kJ/mol with respect to reactants) starting from MIN3. Two barrierless H-displacement channels can be observed starting from MIN4. In particular, the fission of the N–H bond leads to the formation of H + CH_3_CCN (channel d) (the resulting exothermicity of this channel is 142 kJ/mol), while the fission of a C–H bond in the CH_3_ group leads to a production of H + H_2_CCCNH (channel j) (the resulting channel exothermicity is 265 kJ/mol). Moreover, the intermediate MIN4 can undergo a C–C bond breaking with the formation of CH_3_ + linear-CCNH (channel b). MIN6 can undergo different fission processes. The breaking of a C–C bond allows the formation of CN + CH_3_CH (channel a) in a barrierless process (the reaction enthalpy is −45 kJ/mol). The fission of a C–H bond can form CH_3_CCN + H (channel d), also resulting from the dissociation of MIN4, while the breaking of the C–C bond in the CH_3_ group can form CH_3_ + HCCN (channel e) (the resulting exothermicity is 162 kJ/mol). A barrier (TS5) of 209 kJ/mol must be overcome for forming MIN7, a second isomer, which shows the presence of a CN group. This species, which is located 616 kJ/mol below the reactants, can produce CN + C_2_H_4_ (ethylene) (channel l). The elimination of H, together with the formation of acrylonitrile (H_2_CCHCN) (channel n), is characterized by the presence of a barrier (TS9) of 194 kJ/mol (from MIN7). The related transition state TS9 shows the increasing of the CH bond length up to 1.987 Å. One of the last isomers identified along the potential energy surface (MIN8) is formed starting from MIN7, through a barrier (TS7) of 235 kJ/mol. MIN8 can also lead to the formation of H_2_CCHCN (cyanoethylene, also known as acrylonitrile) (channel n) via TS11. Moreover, the breaking of a C–C bond can produce HCN + H_2_CCH (with an exothermicity of 428 kJ/mol) (channel m). The related transition state TS10 is located 413 kJ/mol under the energy of the reactants and exhibits a distance between the two C atoms of 2.287 Å. Additional H shift processes, starting from MIN8, can lead to the formation of MIN9 and MIN10, located, respectively, 560 and 516 kJ/mol below the energy of the reactants. Different H loss processes can be responsible for the formation of several already described species, including H_2_CCHCN (channel n), H_2_CCCNH (channel j), and HCCCHNH (channel k). All the identified stationary points lie under the reactant energy asymptote. In Figures 5–8 the geometries (in Å) of the different minima and products identified along the PES, together with the main saddle points, optimized at the B3LYP/aug-cc-pVTZ level of theory, are reported.

### 4.2 Kinetic Calculations

Kinetic calculations were performed considering four different values of the total energy (0.8, 1.5, 2.5, and 31 kJ/mol) corresponding, respectively, to the most probable collision energy at the surface temperature of Titan (94 K), its stratospheric temperature (175 K), room temperature (298 K), and the collision energy of the present CMB experiment. The results of electronic structure calculations show two different initial steps for the reaction: (i) N(^2^D) can attack the triple bond of the hydrocarbon molecule, forming a cyclic intermediate, or (ii) competitively insert into one of the C–H bonds of the CH_3_ group. We performed RRKM calculations by considering the two initial insertion and addition intermediates separately. Subsequently, we merged the results of the two separate schemes into a global one where the insertion and addition mechanisms were partitioned as detailed in section 2.3. In Table 1 are reported the BFs obtained at a total energy of 31.0 kJ/mol (*E*_c_ of the CMB experiment) for the two possible mechanisms, insertion and addition, separately. For the addition of N(^2^D) to the triple bond of CH_3_CCH, the dominant channel (BF = 56.9%) is a CH_3_-loss channel accompanied to the formation of a cyclic radical (channel g). On the one hand, the second most abundant channel (BF = 21.1%) is a H-displacement one accompanied to the formation of CH_2_CHCN (channel n). On the other hand, the insertion of N(^2^D) into the C–H bond can lead to the formation of two different products. In particular, the barrierless breaking of a C–C bond in MIN5 can lead to the formation of CH_2_NH + CCH (channel h), with a BR of 94.7%, while the remaining 5.3% is associated with another H-displacement channel leading to the formation of CHCCHNH (channel k). The preference for the C–C bond-breaking channel can be explained by the presence of a high barrier for the H elimination process of 175 kJ/mol.

**Table 1:** Branching Fractions (BF) (%) of All Possible Reaction Channels for the Reaction N(^2^D) + CH_3_CCH from Addition Pathways and Insertion Pathways at Total Energy of 31.0 kJ/mol (See Text)

reaction channel	products	addition (MIN1) BF (%)	insertion (MIN5) BF (%)
a	CH_3_CH + CN	0.0%	
b	CCNH + CH_3_	0.1%	
c	c-H_2_CC(NH)C + H	0.4%	
d	CH_3_CCN + H	0.9%	
e	HCCN + CH_3_	1.5%	
f	c-CH_3_C(N)C + H	6.4%	
g	c-C(N)CH + CH_3_	56.9%	
h	CH_2_NH + CCH		94.7%
i	c-CH_2_C(N)CH + H	7.5%	
j	CH_2_CCNH + H	3.8%	
k	CHCCHNH + H		5.3%
l	C_2_H_4_ + CN	1.2%	
m	C_2_H_3_ + HCN	0.02%	
n	CH_2_CHCN + H	21.1%	

The global BFs for the N(^2^D) + CH_3_CCH reaction, obtained as described in section 2.3, are shown in Table 2. Within the frame of our approximate method, the main reaction channel is the one associated with the insertion mechanism and subsequent fission of the C–C bond, leading to the formation of methanimine (CH_2_NH) + CCH (ethylidyne) (channel h). The BF under the conditions of the CMB experiments is 41.4%. The second most important channel (BF = 32.0%) is associated with the addition mechanism and subsequent CH_3_ elimination with formation of the cyclic cofragment *c*-C(N)CH (channel g). The third most important channel is a H-displacement channel associated with acrylonitrile formation, with a BF of 11.9% (channel n). This can be explained considering the high exothermicity of this channel and the three main possible formation routes, respectively, starting from MIN6, MIN7, and MIN8, with that starting from MIN6 having the lowest exit barrier (TS8) (−434 kJ/mol with respect to reactants) (the others are at −422 and −415 kJ/mol, respectively). MIN9 and MIN10, because of the high barriers (TS16 and TS17) to reach them from MIN8, contribute negligibly, with respect to other intermediates, to the calculated BFs of the products that can be reached from them. The formation of other species, such as CN and HCN (channels m and l, respectively), represents only 0.01% and 0.7%, respectively, of the global BFs, while the sum of all (seven in total) possible H displacement processes (channels c, d, f, i, j, k, and n) gives a total value of BF of 24.9%, making the overall H-displacement channels the third most important product channel mechanism in the global reaction.

**Table 2:** Global Branching Fractions (%) for All Possible Reaction Channels for the Reaction N(^2^D) + CH_3_CCH at Different Values of Total Energy (See Text)

reaction channel	products	0.8 kJ/mol	1.5 kJ/mol	2.5 kJ/mol	31.0 kJ/mol
a	CH_3_CH + CN	0.0%	0.0%	0.0%	0.0%
b	CCNH + CH_3_	0.02%	0.02%	0.02%	0.04%
c	c-H_2_CC(NH)C + H	0.1%	0.1%	0.1%	0.2%
d	CH_3_CCN + H	0.5%	0.5%	0.5%	0.5%
e	HCCN + CH_3_	0.9%	0.9%	0.9%	0.9%
f	c-CH_3_C(N)C + H	2.9%	3.0%	3.1%	3.6%
g	c-C(N)CH + CH_3_	27.7%	28.1%	29.0%	32.0%
h	CH_2_NH + CCH	41.1%	41.1%	41.2%	41.4%
i	c-CH_2_C(N)CH + H	4.2%	4.2%	4.2%	4.2%
j	CH_2_CCNH + H	2.7%	2.7%	2.6%	2.2%
k	CHCCHNH + H	2.6%	2.5%	2.5%	2.3%
l	C_2_H_4_ + CN	0.9%	0.9%	0.8%	0.7%
m	C_2_H_3_ + HCN	0.01%	0.01%	0.01%	0.01%
n	CH_2_CHCN + H	16.26%	15.85%	14.99%	11.9%

It is interesting to examine the variation with energy of the BFs for the various channels. The increase of the total energy leads to an increase of the BFs of the dominant CCH + CH_2_NH channel (h) from 41.1% at 94 K up to 41.4% at the *E*_c_ of the CMB experiment (a very modest variation), while for the second most important channel (g) the BF increases (more significantly) from 27.7% to 32.0%. Conversely, the value of the BF calculated for the case of the most exothermic of all H channels, namely, channel (n) forming H + H_2_CCHCN, drops significantly from 16.3% at 94 K (0.8 kJ/mol) to 11.9% at the *E*_c_ of the CMB experiment. Overall, the results in Table 2 show that there is little dependence on the energy available to the system.

## 5 Discussion

The PES of Figure 1 forms the basis of the discussion. The best-fit CM product angular and translational energy distributions displayed in Figure 4a,b and the BFs reported in Table 2 allow an evaluation of the dynamical influence of the PES and of kinematic constraints. As already mentioned, experimentally we were able to probe and characterize only the H-displacements channels; however, theoretically, via RRKM statistical calculations on the computed PES, we provided an estimate of the BFs of all 14 examined exothermic reaction channels.

The experimental data clearly demonstrate that one or more H-displacement channels are occurring. According to our electronic structure calculations, seven isomers with the gross formula of C_3_H_3_N can be formed (see sections 1 and 2.2). A satisfactory fit of the LAB angular distribution and TOF spectra can be achieved by using a single set of CM functions, which implies that our data are not sensitive enough to allow a disentanglement of the possible different contributions that originate from more than one reaction channel. The high-energy cutoff of the best-fit *P*(*E*′_T_) is at 455 ± 20 kJ/mol (Figure 4b). Once the contribution made by *E*_c_ is accounted for, this value is an indication that the most exothermic H-displacement channels is formed. A contribution from the other H-displacement channels, however, cannot be ruled out.

We recall that the CM product angular distribution, *T*(θ), contains information about the micromechanism of the reaction; that is, its shape tells us whether the reaction is direct (i.e., it occurs on the time scale of molecular vibrations) or proceeds via the formation of a long-lived complex intermediate^73−75^ (i.e., it occurs on the time scale of several molecular rotations), while the product translational energy distribution, *P*(*E*′_T_), is determined by the characteristics of the PES (for instance, it could arise from the postbarrier dynamics in the exit channel) and provides a measure of the product energy partitioning between translational and internal (ro-vibrational and electronic) degrees of freedom. As already noted in section 3 the symmetric *T*(θ) (Figure 4a) indicates that the formation of C_3_H_3_N isomeric products proceeds through a long-lived complex mechanism.^73−75^ This is fully supported by our calculations of the PES, which is characterized by bound intermediates associated with deep wells along the possible reaction pathways (see Figure 1).

Next, we attempt to discern the contributions from the various constitutional isomeric products of gross formula C_3_H_3_N. The extent of the energy release, revealed by the shape of the *P*(*E*′_T_) (Figure 4b), provides us a criterion (through the energy conservation rule^75^) to establish which products of general formula C_3_H_3_N are possible. The *P*(*E*′_T_) cutoff defines the maximum available energy of the products, and the vertical lines represented in Figure 4b indicate the total available energy for the five most exothermic isomeric channels of interest, whose heavy detected coproducts have gross formula C_3_H_3_N. Clearly, the *P*(*E*′_T_) is consistent with the strongly exothermic CH_2_CHCN + H channel (n) (Δ*H*^0^_0_ = −438 kJ/mol), that is, with the formation of acrylonitrile (cyanoethylene). In fact, the *P*(*E*′_T_) cutoff of 455 ± 20 kJ/mol nicely matches the total available energy for this channel (*E*_tot_ = Δ*H*^0^_0_ + *E*_c_ = (438 ± 6) + (31 ± 2) = 469 ± 8 kJ/mol), and therefore the presence of this product is ascertained. The other possible isomer products are characterized by a total energy that is lower than that foreseen by the *P*(*E*′_T_) cutoff, and it is not possible to exclude their presence solely from these data, as the sensitivity of the method is such that the achievement of the best fit would have been possible also using several contributions (one for each isomeric channel), but the determination of their relative weight would have not been reliable. In fact, the use of a multicontribution analysis algorithm^45^ is justified only when it is not possible to reach the best fit with a single contribution.

As can be seen from Table 2, the formation of CH_2_CHCN (channel n) is predicted to be the dominant H-displacement channel (BF = 11.9%). From the PES, the minima that can lead to the acrylonitrile-forming channel are mainly MIN6, MIN7, and MIN8, which are, respectively, at −662, −616, and −583 kJ/mol with respect to the reagents and 224, 178, and 145 kJ/mol below the products H_2_CCHCN + H. The *P*(*E*′_T_) distribution peaks at ∼46 kJ/mol. If we refer to the most exothermic channel (n) leading to CH_2_CHCN + H, 33% of the total available energy (indicated by a vertical line in Figure 4b) is released into the product translational energy, suggesting relatively loose exit transition states (TS8, TS9, and TS11 in Figure 1) and the formation of highly internally excited products.

The RRKM results (see Table 2) predict that the overall BF of the seven H-displacement channels is 24.9%. Of these, channel (n), with its BF of 11.9%, represents ∼50% of the H-forming yield. The second most important H channel is channel (i) forming *c*-CH_2_C(N)CH from MIN1 (BF = 4.2%), followed by channel (f) (CH_3_C(N)C + H) (BF = 3.6%), and by channels (j) and (k) forming linear isomers (CHCCHNH and CH_2_CCNH, respectively) with BF = 2.3% and 2.2%. The last two, less exothermic H channels (c) and (d) (not indicated in Figure 4b) are predicted to be minor (BF = 0.2% and 0.5%, respectively).

The statistical calculations indicate that, at the energy of the CMB experiment, the most abundant product channels of the N(^2^D) + CH_3_CCH reaction are channels (g) and (h), leading to *c*-C(N)CH + CH_3_ and CH_2_NH + CCH, with BFs of 32.0% and 41.4%, respectively. Channel (h) (Δ*H*^0^_0_ = −204 kJ/mol) is the main, most favored product channel from the barrierless unimolecular decomposition of the linear intermediate MIN5 formed by N(^2^D) *insertion* into the CH bond of the CH_3_ group of CH_3_CCH. Instead, channel (g) (Δ*H*^0^_0_ = −185 kJ/mol) is the main, most favored product channel from the barrierless unimolecular decomposition of the cyclic intermediate MIN1 formed by the N(^2^D) *addition* to the triple bond of CH_3_CCH. Notably, the BF of both channels exhibits a small energy dependence, especially channel (h) (see Table 2). These results indicate that a CH_3_ loss from the initially formed MIN1 intermediate, reached by an addition of N(^2^D) to the triple bond, with the formation of *c*-HC(N)C, is highly favored (BF = 32.0%) with respect to the formation of linear HCCN (channel e) (BF = 0.9%) and also with respect to H loss with the formation of *c*-CH_2_C(N)CH (BF = 4.2%) (channel (i)). In competition with the above three channels, MIN1 can also isomerize along two main pathways going to MIN3 and MIN4. MIN 4 can lead barrierlessly to CH_2_CCNH + H (channel j) with BF = 2.2%. Instead, MIN3 can isomerize to MIN6, which, via a loose-exit TS8, leads to the main H-forming channel (n) (CH_2_CHCN + H) (BF = 11.9%). The latter product channel can also be formed following a further isomerization of MIN6 to MIN7 and MIN8. In summary, the high probability of losing a CH_3_ group by MIN1 and a C_2_H group by MIN5 with respect to other pathways is the reason why the two channels g and h are predicted to account for ∼73% of the total reaction yield. Unfortunately, as already discussed, the limitations caused by the elastic contaminations impeded us in our effort to characterize the N/CH_3_ and N/CCH exchange channels (g and h, respectively), and the present analysis is limited to the H-displacement channels.

### 5.1 Comparison with N(^2^D) + HCCH (Acetylene)

The interpretation of our experimental findings can be assisted by a comparison with the related N(^2^D) + C_2_H_2_ reaction,^25^ which can be considered as the prototype of the N(^2^D) + alkyne reactions. We expect some analogy in the reaction mechanism because both CH_3_CCH and HCCH contain a C–C triple bond, and both reactions are barrierless. However, while an addition to the triple bond is the only attack site for N(^2^D) in the first step of the N(^2^D) + C_2_H_2_ reaction, for N(^2^D) + CH_3_CCH, in the light of the statistically calculated BFs, it represents only ∼50% of the probability of attack, with the rest being N atom insertion (see above). Notably, the presence of a CH_3_ group removes the symmetry of acetylene, and more reactive channels are open for the title reaction. In our previous study of N(^2^D) + C_2_H_2_ we measured the C_2_HN product (detected at *m*/*z* = 39 and 38) associated with the H-displacement at the *E*_c_ of 13.0 and 39.7 kJ/mol.^25^ The CM angular distribution was isotropic at the lower *E*_c_, while it was slightly forward-biased at the higher *E*_c_. These characteristics are compatible with the formation of one (or more) bound intermediate(s), the lifetime of which is considerably longer than its rotational period at the lower *E*_c_, but they start to osculate with increasing energy because of the shortening of its lifetime (from the anisotropy of the *T*(θ) an average complex lifetime of approximately one rotational period could be estimated at *E*_c_ = 39.7 kJ/mol). It is interesting to note that the *T*(θ) derived for the title reaction at *E*_c_ = 31.0 kJ/mol is consistent with a long-lived complex even though the *E*_c_ is closer to, yet ∼9 kJ/mol lower than, the higher *E*_c_ of the N(^2^D) + C_2_H_2_ experiment. The energies associated with the minima on the C_3_H_4_N PES (Figure 1) are located at values very similar to those associated with the C_2_H_2_N PES (see Figure 6 in ref (25)). Therefore, the increase of the lifetime of the reaction intermediates (which determines the observed long-lived complex mechanism at an *E*_c_ comparable to that of the N(^2^D) + HCCH experiment) for N(^2^D) + CH_3_CCH with respect to those in the analogous N(^2^D) + C_2_H_2_ reaction, is likely caused by the increased number of degrees of freedom among which to distribute the large amount of energy liberated by the formation of the bound intermediates.

It is interesting that an analysis of the PESs for the two related systems highlights how, for N(^2^D) + CH_3_CCH, the overall formation pathways of CH_2_CHCN + H (BF = 11.9%) (rather than CH_3_CCN + H, channel (d), BF = 0.5%) and *c*-CH_2_C(N)CH + H (BF = 4.2%) (rather than *c*-CH_3_C(N)C + H, channel (f), BF = 3.6%) resemble those of linear HCCN + H and *c*-HC(N)C + H, respectively, from N(^2^D) + HCCH. In other words, in the reaction with methylacetylene, the loss of an H atom from the methyl group appears to be favored over the loss of the H atom from the acetylenic carbon. Interestingly, regarding the overall relative yield of H displacement channels, at a comparable *E*_c_, the ratio of linear/cyclic isomers is similar in N(^2^D) + CH_3_CCH (linear/cyclic ≈ 2.1) and N(^2^D) + HCCH (linear/cyclic ≈ 2.7). The presence of the methyl group in CH_3_CCH opens additional H-displacement channel routes, due to the possibility of a migration of the H atom from the CH_3_ group to the acetylenic carbons as well as to the N atom (see Figure 1), that cannot be found in the reaction of N(^2^D) with acetylene. Actually, these chemical pathways are prevalent under our experimental conditions (see the product BFs in Table 2).

### 5.2 Comparison with N(^2^D) + CH_2_CCH_2_ (Allene)

Finally, it is of interest to also compare the reaction dynamics of N(^2^D) + methylacetylene with that of the isomeric reaction N(^2^D) + allene, recently also studied in our laboratory at a comparable *E*_c_ (unpublished results). Although the PESs for the two reactions present some similarities, they also feature dramatic differences. First of all, one does not expect that the product channels of CH_3_ elimination and C_2_H elimination, which are dominant in N(^2^D) + CH_3_CCH, are significant in N(^2^D) + CH_2_CCH_2_. In fact, in the latter reaction the dominant channels are found to be two H-displacement channels, one leading to cyclic *c*-CH_2_C(N)CH + H (BF ≈ 87%) and the other to linear HCCCHNH (propargylimine) + H (BF ≈ 10%), while the formation of acrylonitrile + H is minor (BF < 1%) (unpublished results). These findings are expected to be of considerable relevance for the detailed modeling of Titan’s atmosphere.

## 6 Implications for the Atmosphere of Titan

The reactions between N(^2^D) and methylacetylene (and its isomer allene) have already been included in photochemical models of the atmosphere of Titan with estimated parameters. As already observed in other similar cases, those parameters can differ significantly from the real values as determined by laboratory experiments or theoretical estimates. For instance, in the most recent models^41,42^ the reaction N(^2^D) + methylacetylene has been included with a global rate coefficient of 1.6 × 10^–10^ exp(−270/*T*). As already mentioned in the Introduction, only two channels have been considered (HCCN + CH_3_ and C_2_H_3_CN + H), accounting each for one-half of the global rate coefficient. The reaction N(^2^D) + allene is included with a global rate coefficient of 2.3 × 10^–10^ exp(−503/*T*) and only one possible channel, that is, C_2_H_3_CN + H.

According to the present study, the key characteristics of the reaction between N(^2^D) and CH_3_CCH include: no entrance barrier, energies for all the involved transition states lying below the asymptote of the reactants, very exothermic product channels. Therefore, this bimolecular reaction is expected to be very fast even at low temperatures. Notably, a recent kinetic study of the isomeric variant N(^2^D) + allene found a large value of the rate coefficient (in the 10^–10^ cm^3^/s range) essentially independent of the temperature in the range of 50–300 K (unpublished results). On the contrary, the rate coefficients of N(^2^D) + allene and N(^2^D) + methylacetylene in current photochemical models are assumed to decrease with decreasing temperature and to become of the order of 5 × 10^–12^ cm^3^/s at 100 K.^41,42^ Concerning the main product channel of N(^2^D) + allene, in a study similar to the one presented here, we have established that the BF of acrylonitrile (the only channel considered for the reaction with allene) is much less than 1%, while the dominant channel is another H-displacement channel accompanied by the formation of the *c*-H_2_CC(N)CH radical (unpublished results). We recall that methylacetylene is ∼8 times more abundant than allene on Titan.^16,42^ We emphasize that the reaction N(^2^D) + methylacetylene is assumed to produce HCCN + CH_3_ and CH_2_CHCN + H (channels e and n, respectively, in Table 2) in equal amounts at all temperatures,^41^ while our statistical calculations predict for channel (e) a BF of 0.9% at 175 K and for channel (n) a BF of 15.9% at 175 K (see Table 2). Clearly, a refinement of models with the inclusion of the correct temperature dependence of the global rate constant and the correct product BFs is in order.

Overall, we recommend that the reaction N(^2^D) + methylacetylene is included in photochemical models with a large rate coefficient, of the order or 10^–10^ cm^3^/s even at temperatures below 200 K and down to 50 K, with the main products being methanimine + C_2_H, *c*-C(N)CH + CH_3_, and acrylonitrile + H. Both methanimine and acrylonitrile have been identified in the upper atmosphere of Titan. And even though other formation routes have been envisaged for both of them (namely, the reactions N(^2^D) + CH_4_ and N(^2^D) + C_2_H_6_, with the addition of the reaction NH + CH_3_ and NH + C_2_H_5_ for methanimine^76,77^ and the reaction CN + C_2_H_4_ for acrylonitrile^78−80^), the title reaction can provide an additional source. Methanimine is already overpredicted in the photochemical models, and the additional contribution of the title reaction will worsen the comparison. This adds to other previous suggestions that destruction routes of methanimine are missing in the photochemical models, especially after the possibility of a spontaneous isomerization has been ruled out.^81^

A more general conclusion is that we have verified that also the title reaction leads to molecules holding a new C–N bond, such as, for instance, methanimine (CH_2_NH) (BF ≈ 41%), *c*-C(N)CH (BF ≈ 28%) (the same product is also formed from the N(^2^D) + HCCH reaction^25^), and acrylonitrile (cyanoethylene) (CH_2_CHCN) (BF ≈ 16%). These species may have a potentially significant impact on the gas-phase chemistry of Titan’s atmosphere. We recall that imines are key intermediates in the formation of biologically important precursors such as nitrogenous bases and amino acids, which are the main building blocks of nucleic acids and proteins.^82^

## 7 Conclusions

The N(^2^D) reaction with methylacetylene was investigated by means of the CMB technique with mass spectrometric detection at the collision energy of 31.0 kJ/mol and electronic structure calculations of the underlying potential energy surface. The angular and TOF distributions of C_3_H_3_N products in the LAB frame along with the derived CM best-fit functions suggest that the reaction mechanism features the formation of one or more C_3_H_4_N intermediates with a lifetime longer than their rotational periods. The translational energy distribution reveals that C_3_H_3_N products are internally (ro-vibrationally) excited and that the most exothermic of all possible H forming channels, namely, acrylonitrile + H, is being formed, with also other isomers of acrylonitrile being possible. Synergistic RRKM statistical calculations on the ab initio doublet C_3_H_4_N PES of product distributions and branching fractions corroborate and complement our findings for the H-displacement channels and provide a more complete picture of the overall reaction mechanism, with up to 14 competing product channels being open, and for which product BFs are calculated as a function of energy. Of these 14 channels, one-half features a BF less than 1%. Our calculations show that this reaction is initiated, competitively, by both the barrierless *addition* of N(^2^D) atom to the triple bond of CH_3_CCH, forming a cyclic adduct complex *c*-CH_3_C(N)CH (MIN1), and by an *insertion* of N(^2^D) into the CH bond of the methyl group, forming a linear adduct complex HNCH_2_CCH (MIN5) of stability similar to the cyclic one (MIN1). By the breaking of the C–C bond, these intermediates (MIN1 and MIN5) can directly dissociate predominantly to C(N)CH + CH_3_ and C(N)CH + CCH, respectively, with a predicted BF of ∼32% and 41%, respectively. MIN1 can also competitively isomerize to a variety of linear complexes of which some lead to the third most important product channel CH_2_CHCN (cyanoethylene, also named acrylonitrile) + H with BF ≈ 12%. Other H-displacement channels are also predicted to occur with BF ranging from 4.2% to 0.2%, for a total BF of the seven predicted H channels of 24.9%.

Our studies provide the first evidence that the reaction of N(^2^D) with CH_3_CCH is a potential pathway to produce, in the conditions of the atmosphere of Titan, methanimine (41%), c-C(N)CH (28%), and acrylonitrile (16%) in the gas phase (Table 2, *E* = 1.5 kJ/mol), which in turn can further react efficiently with other species acting as precursors of other nitriles (C_2_N_2_, C_3_N) or more complex organic molecules containing a CN bond by consecutive reactions.

In conclusion, our findings on the title reaction could be incorporated into photochemical models of N_2_-rich planetary atmospheres (in particular, of Titan) bearing a significant amount of unsaturated hydrocarbons, and they might also contribute to a reevaluation of the role of gas-phase neutral chemistry in heavily UV-irradiated interstellar environments that contain N_2_ and hydrocarbons, such as comets, where the main fate of N(^2^D) is considered to be collisional quenching,^83^ while the main reaction of N(^2^D) in comets appears to be that with H_2_O (water).^84,85^

## Supporting Information Available

Reaction enthalpies and barrier heights (kJ/mol, 0 K) computed at the CCSD(T)/aug-cc-pVTZ level of theory, considering the geometries obtained at the B3LYP/aug-cc-pVTZ level, for dissociation and isomerization processes for the system N(^2^D) + CH_3_CCH (PDF)
