# Supplementary material for: The Reaction N(2D) + CH3CCH (Methylacetylene): A Combined Crossed Molecular Beams and Theoretical Investigation and Implications for the Atmosphere of Titan
Source: J Phys Chem A. 2021 Oct 5;125(40):8846–59. doi: 10.1021/acs.jpca.1c06537 (PMC8521525; doi:10.1021/acs.jpca.1c06537)
Supplement: Supplementary file 1 — jp1c06537_si_001.pdf [file jp1c06537_si_001.pdf]

Electronic Supporting Information for the paper

The Reaction $N(^2D)$ + CH_3CCH (Methylacetylene): A Combined Crossed Molecular Beams and Theoretical Investigation and Implications for the Atmosphere of Titan

Luca Mancini,[a] Gianmarco Vanuzzo,[a] Demian Marchione,[a] Giacomo Pannacci,[a] Pengxiao Liang,[a] Pedro Recio,[a,†] Marzio Rosi,[b] Dimitrios Skouteris,[c] Piergiorgio Casavecchia,[a*] and Nadia Balucani[a*]

[a] *Dipartimento di Chimica, Biologia e Biotecnologie, Università degli Studi di Perugia, Perugia, Italy*
[b] *Dipartimento di Ingegneria Civile e Ambientale, Università degli Studi di Perugia, Perugia, Italy*
[c] *Master-Tec srl, Via Sicilia 41, 06128 Perugia, Italy*

This file contains: Table S1. Reaction enthalpies and barrier heights.

Table S1. Reaction enthalpies and barrier heights (kJ/mol, 0 K) computed at the CCSD(T)/aug-cc-pVTZ level of theory considering the geometries obtained at the B3LYP/aug-cc-pVTZ level for dissociation and isomerization processes for the system N(^{2}D) + CH_3CCH.

	ΔH^0_0	Barrier heights
N(^{2}D) + CH_3CCH → MIN1	−446	
N(^{2}D) + CH_3CCH → MIN5	−445	
MIN1 → MIN2	98	140
MIN2 → MIN3	−109	33
MIN2 → MIN4	−178	64
MIN3 → MIN6	−205	23
MIN6 → MIN7	46	209
MIN7 → MIN8	33	235
MIN1 → H + *c*-$CH_3C(N)C$	280	
MIN1 → CH_3 + *c*-C(N)CH	261	
MIN1 → H + *c*-$CH_2C(N)CH$	226	
MIN2 → H + *c*-$CH_2C(NH)C$	258	
MIN2 → H + *c*-$CH_3C(N)C$	182	191
MIN5 → H + HCCCHNH	151	175
MIN5 → CCH + CH_2NH	241	
MIN3 → H + *c*-$CH_3C(N)C$	291	310
MIN3 → CH_3 + *c*-C(N)CH	272	275
MIN4 → CH_3 + CCNH	461	
MIN4 → H + CH_3CCN	384	
MIN4 → H + CH_2CCNH	261	
MIN6 → CN + CH_3CCH	617	
MIN6 → H + CH_3CCN	520	
MIN6 → CH_3 + HCCN	500	
MIN6 → H + CH_2CHCN	224	228
MIN7 → H + CH_2CHCN	178	194
MIN7 → CN + CH_2CH_2	242	

MIN8 → HCN + CH_2CH	155	170
MIN8 → H + CH_2CHCN	145	168
MIN8 → MIN9	23	213
MIN9 → MIN10	44	258
MIN9 → CH_2CHCN + H	122	146
MIN9 → CH_2CCNH + H	295	
MIN10 → CHCCHNH + H	222	236
MIN10 → CH_2CCNH + H	251	254
